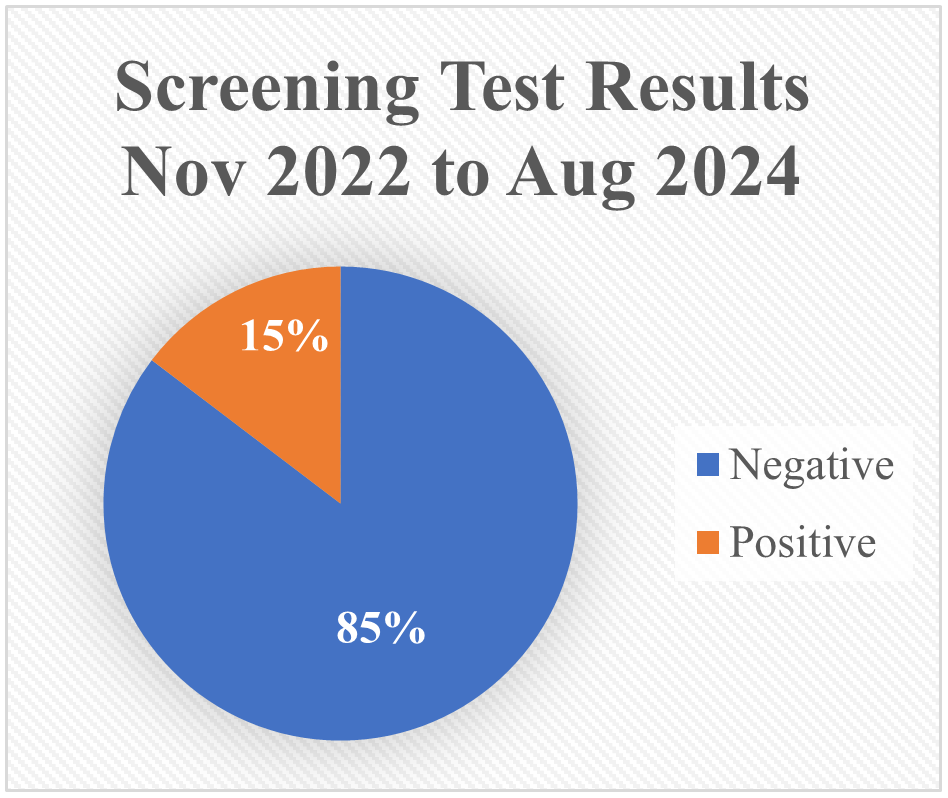# 628 Acute Stress Disorder Screening Tool

**DOI:** 10.1093/jbcr/iraf019.257

**Published:** 2025-04-01

**Authors:** Matthew Supple, Crystal New, Mary Elizabeth Bilodeau, Deanna McNally, Katherine Kurpaska, Sherriann King, Francisca Alvarez, John Schulz, Jeremy Goverman, Jonathan Friedstat, Sean Hickey, Nadia Quijije, Mladen Nisavic

**Affiliations:** Massachusetts General Hospital; Massachusetts General Hospital; Massachusetts General Hospital; Massachusetts General Hospital; Massachusetts General Hospital; Massachusetts General Hospital; Massachusetts General Hospital; Massachusetts General Hospital; Massachusetts General Hospital; Massachusetts General Hospital; Massachusetts General Hospital; Massachusetts General Hospital; Massachusetts General Hospital

## Abstract

**Introduction:**

Universal screening for acute stress disorder (ASD) is recommended by the American Burn Association (ABA) but can be challenging to implement in U.S. burn centers due to lack of resources and visit time constraints. Recent studies have shown the prevalence of ASD in burn patients can range from 1% up to 30%. We aimed to formalize our ASD screening process in a way that allowed time for patients to privately self-survey during their visit.

**Methods:**

By working with a burn-specific psychiatrist a patient- led questionnaire assessing nightmares, flashbacks, sleep hygiene, and social activities was formulated. Positive answers to 3 or more questions indicated the presence of acute stress disorder, triggering a referral to our psychiatrist. This original survey was used at three intervals post burn injury. Those that screened negative at two weeks, were found not to screen positive at later assessments. Ultimately it was determined the diagnosis of ASD was made two weeks after burn injury. We eliminated screening at the four- and twelve-week interval.

**Results:**

A total of 611 patients were screened for ASD during their outpatient clinic follow-up visits from November 2022 until August 2024. Out of these 611 individuals, 87 individuals screened positive. Of those 87 positive screens, 21 individuals accepted the referral with our psychiatrist, 11 individuals already had a therapist/ psychiatrist or would seek a therapist elsewhere, and the remaining 55 individuals declined any intervention. The average age of those individuals was 47 years old and had an average TBSA burn of 3.5%. Additionally, the mechanism of injury varied widely among patients that screened positive. The most common burn etiology was scald injuries, accounting for 55% of the injuries.

**Conclusions:**

ASD is a common response that can occur after a traumatic incident like burn injuries. With 15% of all burn patients seen since November 2022 screening positive, it has become increasingly important to screen all patients that come through our clinic. Early identification of ASD allows for early intervention, and the potential to mitigate the long-term psychological effects of a burn injury.

**Applicability of Research to Practice:**

This tool can be easily administered in an inpatient or outpatient clinic setting and completed independently.

**Funding for the Study:**

The authors received no external funding for this research.